# Immune responses to Sinopharm/BBIBP‐CorV in individuals in Sri Lanka

**DOI:** 10.1111/imm.13536

**Published:** 2022-07-12

**Authors:** Chandima Jeewandara, Inoka Sepali Aberathna, Pradeep Darshana Pushpakumara, Achala Kamaladasa, Dinuka Guruge, Ayesha Wijesinghe, Banuri Gunasekera, Shyrar Tanussiya, Heshan Kuruppu, Thushali Ranasinghe, Shashika Dayarathne, Osanda Dissanayake, Nayanathara Gamalath, Dinithi Ekanayake, Jeewantha Jayamali, Deshni Jayathilaka, Madushika Dissanayake, Deshan Madusanka, Tibutius Thanesh Jayadas, Anushika Mudunkotuwa, Gayasha Somathilake, Michael Harvie, Thashmi Nimasha, Saubhagya Danasekara, Ruwan Wijayamuni, Lisa Schimanski, Pramila Rijal, Tiong K. Tan, Tao Dong, Alain Townsend, Graham S. Ogg, Gathsaurie Neelika Malavige

**Affiliations:** ^1^ Allergy Immunology and Cell Biology Unit, Department of Immunology and Molecular Medicine University of Sri Jayewardenepura Nugegoda Sri Lanka; ^2^ Colombo Municipal Council Colombo Sri Lanka; ^3^ MRC Human Immunology Unit MRC Weatherall Institute of Molecular Medicine, University of Oxford Oxford UK; ^4^ Centre for Translational Immunology Chinese Academy of Medical Sciences Oxford Institute, University of Oxford Oxford UK

**Keywords:** antibodies, B cell, T cell, vaccination, viral

## Abstract

As there are limited data of the immunogenicity of the Sinopharm/BBIBP‐CorV in different populations, antibody responses against different SARS‐CoV‐2 variants of concern and T cell responses, we investigated the immunogenicity of the vaccine, in individuals in Sri Lanka. SARS‐CoV‐2‐specific antibodies were measured in 282 individuals who were seronegative at baseline, and ACE2 receptor blocking antibodies, antibodies to the receptor‐binding domain (RBD) of the wild‐type (WT), alpha, beta and delta variants, ex vivo and cultured IFNγ ELISpot assays, intracellular cytokine secretion assays and B cell ELISpot assays were carried out in a sub cohort of the vaccinees at 4 and 6 weeks (2 weeks after the second dose). Ninety‐five percent of the vaccinees seroconverted, although the seroconversion rates were significantly lower (*p* < 0.001) in individuals >60 years (93.3%) compared to those who were 20–39 years (98.9%); 81.25% had ACE2 receptor blocking antibodies at 6 weeks, and there was no difference in these antibody titres in vaccine sera compared to convalescent sera (*p* = 0.44). Vaccinees had significantly less (*p* < 0.0001) antibodies to the RBD of WT and alpha, although there was no difference in antibodies to the RBD of beta and delta compared to convalescent sera; 27.7% of 46.4% of vaccinees had ex vivo IFNγ and cultured ELISpot responses respectively, and IFNγ and CD107a responses were detected by flow cytometry. Sinopharm/BBIBP‐CorV appeared to induce a similar level of antibody responses against ACE2 receptor, delta and beta as seen following natural infection.

## Graphical abstract

Ninety‐five percent of individuals appear to seroconvert following the Sinopharm/BBIBP‐CorV vaccine, and the vaccine appears to induce similar levels of ACE2 receptor blocking antibodies and RBD binding antibodies to delta variant as seen following natural infection; 27.7% of individuals had ex vivo IFNγ ELISpot responses to the vaccine, while CD107a expression was predominantly seen from the CD4^+^ subset of T cells. However, seroconversion rates and immunogenicity appear to be lower in older individuals.

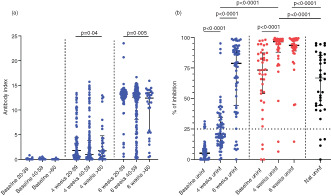

## INTRODUCTION

Although the COVID‐19 pandemic has caused unprecedented mortality and morbidity in almost all countries in the world, within a period of 1 year, several vaccines for COVID‐19 have been developed. The mRNA vaccine Pfizer BioNTech (BNT162b2) was the first to receive emergency use authorization by the WHO in December 2020, followed by many other vaccines such as AZD1222 (AstraZeneca), Jansen Ad26, COV2.S, Moderna mRNA1273, Sinopharm/BB1BP‐CorV and Sinovac [1]. During this short time, many of these vaccines have been extensively studied, including the persistence of neutralization antibody responses, antibody responses to SARS‐CoV‐2 variants of concern (VOCs), memory T cell and B cell responses, memory T cell and B cell repertoire [2, 3, 4]. However, there are limited data regarding the immune responses of the Sinopharm/BBIBP‐CorV in real‐world situations, memory B cell and T cell responses and responses to SARS‐CoV‐2 VOCs.

Sinopharm/BBIBP‐CorV is an inactivated Vero‐cell derived whole‐virus vaccine, which demonstrated high levels of neutralizing antibodies in animal models and prevented infection in animal challenge models [5]. The vaccine was also shown to be well tolerated and 79%–96% individuals were shown to seroconvert at 14 days after the second dose, while all individuals (100%) were shown to seroconvert by 28 days. All individuals in the vaccine arm were reported to have neutralizing antibodies by 42 days, following the second dose of the vaccine [6]. The phase 3 demonstrated an efficacy of 78.1% against symptomatic illness [7], and it was given emergency user licence by the WHO on 7 May 2021. Sinopharm/BBIBP‐CorV is the predominant vaccine used in many Asian and Middle East countries and was recently included in the Global Alliance for Vaccines and Immunizations, to be distributed under the COVAX program [8].

Although safety, immunogenicity and efficacy data for Sinopharm/BBIBP‐CorV are available, there are limited data on immunogenicity in different populations, and there are limited data regarding T cell responses to this vaccine in humans or responses to the emerging VOCs such as B.1.617.2 (‘delta variant’). The related delta variants have been shown to result in immune evasion and escape neutralization by vaccine‐induced neutralization antibodies and by convalescent sera, although to a lesser extent than beta (B.1.351) [9]. However, although VOCs have been shown to escape immunity induced by antibodies, they were less likely to evade T cell immunity [10]. Given that the delta is the dominant variant in many countries and that virus‐specific T cells may play an additional role in protection against severe COVID‐19 [11], it would be important to investigate immunogenicity of this vaccine against VOCs and also to assess virus‐specific T cell responses.

Sinopharm/BBIBP‐CorV is the vaccine most widely used in Sri Lanka and as there are no data regarding its immunogenicity in real‐world situations and to VOCs, we investigated antibody responses to the SARS‐CoV‐2 including to the VOCs, along with T cell responses and their functionality, including memory B cell responses in a large cohort of Sri Lankan individuals.

## METHODS

### Study participants

Three hundred twenty‐three individuals above the age of 21 years, who were vaccinated in Colombo, Sri Lanka, were included in the study following informed written consent. A baseline blood sample was obtained to determine previous SARS‐CoV‐2 infection, at 4 weeks when the second dose has been administered and again 2 weeks from obtaining the second dose of the vaccine (6 weeks from first dose). Presence of comorbid illnesses such as diabetes, hypertension and chronic kidney disease was recorded. Thirty‐six individuals with varying severity of past COVID‐19 were also recruited 6 weeks from the onset of illness, to compare the antibody responses for ACE2 receptor blocking assays and the antibody responses to the VOCs.

Ethics approval was obtained from the Ethics Review Committee of the University of Sri Jayewardenepura.

### Patient characteristics

Twenty patients confirmed with SARS‐CoV2 infection based on the positive RT‐PCR who were treated at a COVID‐19 treatment hospital, were recruited 6 weeks after onset of illness, following informed written consent. Severity of clinical illness and details of laboratory test results were retrieved from the diagnosis card issued by hospitals when the patients were discharged from hospital. Clinical disease severity was classified as mild, moderate and severe according to the WHO guidance on COVID‐19 disease severity [12]. Based on the WHO COVID‐19 disease classification, 32 patients had mild illness and 4 patients had moderate/severe illness.

### Detection of SARS‐CoV‐specific total (IgG, IgM and IgA) antibodies

Seroconversion rates to the BBIBP‐CorV vaccine were determined by using the Wantai SARS‐CoV‐2 antibody ELISA (Beijing Wantai Biological Pharmacy Enterprise), which detects IgM, IgG and IgA antibodies to the receptor‐binding domain (RBD) of the SARS‐CoV‐2. A cut‐off value for each ELISA was calculated according to manufacturer's instructions. Based on the cut‐off value, the antibody index (used as an indirect indicator of the antibody titre) was calculated by dividing the absorbance of each sample by the cut‐off value, according to the manufacturer's instructions.

### ACE2 receptor blocking antibodies

Due to the non‐availability of biosafety level 3 facilities to carry out live neutralization assays a surrogate virus neutralization test (sVNT) [13], which measures the percentage of inhibition of binding of the RBD of the S protein to recombinant ACE2 [13] (Genscript Biotech) was carried out according the manufacturer's instructions as previously described by us [14]. Inhibition percentage ≥25% in a sample was considered as positive for NAbs.

### Haemagglutination tests for detection of antibodies to the RBD in wild‐type and SARS‐CoV‐2 variants

The HAT was carried out as previously described using the alpha (N501Y), beta (N501Y, E484K, K417N) and delta variant (L452R, T478K) versions of the IH4‐RBD reagents [15], which included the relevant amino acid changes introduced by site directed mutagenesis [16]. The assays were carried out and interpreted as previously described [17]. Briefly, sera were doubling‐diluted in 50 μl phosphate‐buffered saline (PBS) in V bottomed 96‐well plates, 50 μl of ~1% vol/vol O negative red cells were added, followed by 50 μl of the relevant IH4‐RBD reagent diluted to 2 μg/ml (100 ng/well). Plates were incubated for 1 h at room temperature, tilted for ~20 s to allow a red cell ‘teardrop’ to form, photographed and read by eye. The RBD‐specific antibody titre for the serum sample was defined by the last well in which the complete absence of ‘teardrop’ formation was observed. The HAT titration was performed using seven doubling dilutions of serum from 1:20 to 1:1280, to determine presence of RBD‐specific antibodies. A titre of 1:20 was considered as a positive response, as previously determined by us [18]. The IH4‐RBD reagents for each VOC were standardized by titration with the monoclonal antibody EY‐6A [15, 16] that binds to a conserved epitope common to all variants. A 20 μg/ml solution of EY‐6A titrated equally with a standard (2 μg/ml) solution of each of the new IH4‐RBD reagents. All were therefore added as 50 μl from a 2 μg/ml stock solution (100 ng/well) as described [15].

### Ex vivo and cultured ELISpot assays

Ex vivo IFNγ ELISpot assays were carried out using freshly isolated peripheral blood mononuclear cells (PBMC) obtained from 66 individuals. Individuals for T cell assays were randomly recruited from the study participants, and we included those who consented to provide an additional blood volume for T cell assays (7 ml), in addition to the antibody assays (5 ml). For cultures ELISpot assays, PBMC from each donor were incubated with the peptides covering the whole spike protein (253 overlapping peptides). Briefly, 5.0 × 10^6^ PBMCs were incubated for 10 days with 200 μl of 40 μM peptide pool in a 24‐well plate. Interleukin‐2 (IL‐2) was added on Day 3 and 7 at a concentration of 100 units/ml. All cell lines were routinely maintained in RPMI‐1640 supplemented with 2 mM l‐glutamine, 100 IU/ml penicillin and 100 μg/ml + 10% human serum at 37° celsius, in 5% CO_2_ [19].

For cultured ELISpot assays, two pools of overlapping peptides named S1 (peptide 1–130) and S2 (peptide 131–253) covering the whole spike protein (253 overlapping peptides) were added at a final concentration of 10 μM and incubated overnight as previously described [20, 21]. For ex vivo ELISpot assays, 100 000 cells/well were added, while for cultured ELISpot assays, 40 000 cells/well were added. All peptide sequences were derived from the wild‐type (WT) consensus and were tested in duplicate. Phytohaemagglutinin was included as a positive control of cytokine stimulation and media alone was applied to the PBMCs as a negative control. An example of an ex vivo ELISpot assay for S1, S2, is shown in Figure S1 and for the cultured ELISpot assays, in Figure S2. Detailed methods of ELISpot assays are described in the Supporting Information Methods S1.

Briefly, ELISpot plates (Millipore Corp.) were coated with anti‐human IFNγ antibody and incubated overnight (Mabtech), with the PBMCs and the relevant overlapping peptides of S1 and S2 pools of peptide. The plates were incubated overnight at 37° celsius and 5% CO_2_. The cells were removed, and the plates developed with a second biotinylated Ab to human IFNγ and washed a further six times. The plates were developed with streptavidin‐alkaline phosphatase (Mabtech AB) and colorimetric substrate. The spots were enumerated using an automated ELISpot reader (AID Germany). Background (PBMCs plus media alone) was subtracted and data expressed as number of spot‐forming units (SFU) per 10^6^ PBMCs. A positive response was defined as mean ± 2 SD of the background responses.

### Intracellular cytokine staining

Intracellular cytokine staining (ICS) was carried out in freshly isolated PBMCs. PBMCs were incubated with CD107a FITC (Biolegend) for 30 min in RPMI‐1640 and 10% heat inactivated human serum (Sigma‐Aldrich). Cells were stimulated with overlapping peptides pool of SARS‐CoV‐2 spike protein for 2 h at a concentration of 10 μg/ml before adding monensin (Biolegend) as previously described [22, 23]. The PBMC were incubated for a further 14 h before staining with live/dead fixable aqua dead cell stain (Thermo Fisher Scientific) according to the manufacturer's protocol to exclude dead cells. Then cells were stained with anti‐CD3 APC Cy7 (clone OKT3), anti‐CD8 BV650 (clone SK1) and anti‐CD4 PB (clone OKT4). Then the cells were fixed with fixation buffer and permeabilized with perm wash buffer (Biolegend) and stained for IFNγ APC (clone 4S.B3). Cells were acquired on a BD FACSAria III Cell Sorter using DIVA v8 software (BD Biosciences). For each donor, unstimulated cells were included as a negative control. Flow cytometry data were analysed using FlowJo v.10.7.1 software (FlowJo). Fluorescence minus one controls were used to draw the gates for both CD107a and IFNγ (Figure S3). The proportion of cells with increased membrane expression CD107a or producing IFNγ, was determined by subtracting the expression levels/production levels in the unstimulated wells, from the peptide stimulated wells.

### B cell ELISpot assays

Briefly, freshly isolated PBMCs were stimulated in a 24‐well plate using IL‐2 and R848 (a TLR 7/8 agonist) to study the memory B cell responses as previously described [24, 25]. The cells were stimulated in RPMI supplemented with 10% foetal bovine serum, 1% penicillin–streptomycin and 1% glutamine at 4 million cells/well and incubated at 37° celsius with 5% CO_2_ for 3 days. They were then washed and rested overnight and 100 000 cells/well were added. Fifty thousand cells/well were added to the positive control wells. A human IgG ELISpot kit (Mabtech 3850‐2A) was used according to the manufacturer's instructions to quantify IgG‐secreting cells specific to SARS‐COV2 S1, S2 and N recombinant proteins, which were coated at 2 μg/ml in PBS. All experiments were carried out in duplicate and anti‐human IgG monoclonal capture antibodies, was used as a positive control, and media alone as a negative control. The spots were enumerated using an automated ELISpot reader (AID). A positive response was defined as mean ± 2 SD of the background responses. An example of a B cell ELISpot assay for S1, S2 and N recombinant proteins is shown in Figure S4.

### Statistical analysis

The percentage calculations for the analysis were conducted in Microsoft Excel and the 95% confidence intervals (CIs) for each category were calculated using the R software (version 4.0.3) and R‐studio (version 1.4.1106). Pearson *χ* ^2^ association tests were performed at a confidence level of 95% using the R software in order to identify the statistically significant associations of the age categories and the sex of the respondents in the study with the 4 and 6 weeks post‐vaccine antibody results. The differences in antibody responses to the vaccine in uninfected uninfected individuals were assessed by the Wilcoxon matched pairs signed ranked test. The differences in antibody responses at those who were found to be infected at the time of recruitment and uninfected individuals was determined by using the Mann–Whitney test. All tests were two sided. The differences in the antibody titres between different age groups was determined by the Kruskal–Wallis test.

## RESULTS

### Seroconversion rates to Sinopharm/BBIBP‐CorV in different age groups and in those with comorbidities

Immune responses were studied at 4 weeks from the first dose and again 2 weeks from the second dose (6 weeks from the first dose) of the vaccine. Of the 323 individuals, 41 individuals were found to have SARS‐CoV‐2 antibodies at the time of enrollment to the study (at the time of receiving the first dose). Of the 282 individuals who were seronegative, 111 (39.4%) were females. The seropositivity rates at 4 and 6 weeks and the median antibody titers and interquartile ranges of different age groups are shown in Table 1. The overall seroconversion rates were 95.0% at 6 weeks (2 weeks after obtaining the second dose), while the seroconversion rates were highest in the 20–39 age group (98.88%). Seroconvserion rates were significantly different in the three age groups (Pearson *χ* ^2^ = 842.983, *p* < 0.001). The seroconversion rates were also significantly higher (Pearson *χ* ^2^ = 836.750, *p* < 0.001) in males (96.5%, 95% CI: 93.73%–99.25%) compared to females (92.83%, 95% CI: 87.98%–97.60%). Forty‐eight (17.0%) individuals had comorbidities (diabetes, hypertension of chronic kidney disease) and there was no difference in seroconversion rates in those with comorbidities (91.72%, 95% CI: 83.83%–99.54%) compared to those without (95.72%, 95% CI: 93.14%–98.31%).

**TABLE 1:** Seroconversion rates and SARS‐CoV‐2 specific total antibody levels (indicated by antibody index) in individuals who received the Sinopharm/BBIBP‐CorV vaccine at 4 and 6 weeks

Age group	Seropositivity at 4 weeks *N* (%), 95% confidence interval	Antibody index (antibody titre) at 4 weeks median, IQR	Seropositivity at 6 weeks *N* (%)	Antibody index (antibody titre) at 6 weeks median, IQR
21–40 *N* = 89	54 (60.67%) (50.53%, 70.82%)	1.79 (0.58–4.48)	88 (98.88%) (96.69%, 100%)	13.22 (12.47–13.72
41–60 *N* = 163	82 (50.31%) (42.63%, 57.98%)	1.0 (0.13–3.85)	152 (93.25%) (89.40%, 97.10%)	12.6 (8.09–13.52)
>60 *N* = 30	19 (63.33%) (46.09%, 80.58%)	1.75 (0.55–4.77)	28 (93.33%) (84.41%, 100%)	12.42 (5.41–13.52)

### Generation of ACE2 receptor blocking antibodies in naïve and previously infected individuals

**FIGURE 1:**
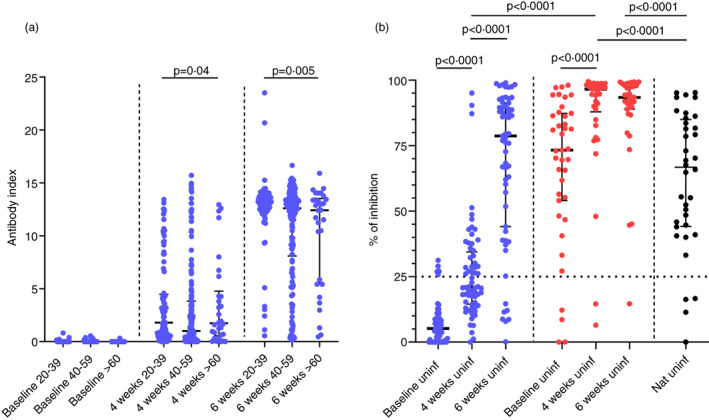
SARS‐CoV‐2 specific antibody responses (total antibodies and ACE2 receptor blocking antibodies in those who received the Sinopharm/BBIBP‐CorV vaccine. (a) SARS‐CoV‐2 specific total antibodies were measured by ELISA in 20‐ to 39‐year‐old individuals (*n* = 89), 40‐ to 59‐year olds (*n* = 163) and >60 years olds (*n* = 30) at 4 weeks following the first dose and again at 6 weeks (2 weeks after the second dose). (b) ACE2 receptor blocking antibodies were measured by the surrogate neutralizing antibody assay in a sub cohort of these individuals (*n* = 64) at 4 and 6 weeks, in a cohort of individuals (*n* = 38), who were found to be seropositive for SARS‐CoV‐2 at the time of recruitment and in a cohort of naturally infected individuals 6 weeks following onset of illness (*n* = 36). The differences in the total antibody titres between different age groups was determined by the Kruskal–Wallis test. the differences in ACE2 receptor blocking antibodies at baseline uninfected and infected individuals were assessed by the Wilcoxon matched‐pairs signed ranked test. all tests were two sided. the error bars indicate the median and the interquartile ranges. Baseline uninfected individuals: Blue, baseline infected individuals: Red, naturally infected unvaccinated individuals: Black

ACE2 receptor blocking antibodies were measured in individuals who were found to be non‐infected (*n* = 64) at the time of recruitment (at the time of obtaining the first dose) and those who were baseline infected (*n* = 38) and in a cohort of naturally infected, unvaccinated individuals (*n* = 36). Those who were naturally infected had varying severity of illness 6 weeks prior to recruitment to the study. A percentage of inhibition of antibody binding to the ACE2 receptor of 25% was considered as a positive response as previously described [14]. At 4 weeks, 24 of 64 (37.5%) of uninfected individuals had a positive response, while 52 of 64 (81.25%) had a response at 6 weeks (Figure 1b). Thirty‐two of thirty‐six (88.9%) of naturally infected individuals had a positive response. The antibody levels significantly increased from baseline (median = 5.2, interquartile range [IQR] = 0.3%–8.5% of inhibition) to 4 weeks (median = 20.96, IQR = 14.9–34.4, *p* < 0.0001% of inhibition) to 6 weeks (median = 78.7, IQR = 44.1–91.2, *p* < 0.0001), in non‐infected individuals. There was no significant difference in antibody levels in those who were naturally infected (median = 66.7, IQR = 44.2%–84.9% of inhibition) compared to baseline uninfected vaccinees at the end of 6 weeks (*p* = 0.15). There was no correlation with age and the ACE2 receptor blocking antibodies at 4 weeks (Spearman's *r* = 0.04, *p* = 0.69) or at 6 weeks (Spearman's *r* = −0.21, *p* = 0.10) and there was no difference in the antibody titres of the three different age groups at 4 or 6 weeks (Figure S1).

Of those who were previously infected (seropositive at baseline), 4 of 38 (10.5%) did not have ACE2 blocking antibodies. By 6 weeks, except for one individual, all others had detectable antibodies (Figure 1b). Although the antibody levels significantly increased from baseline to 4 weeks (*p* < 0.0001), there was no significant increase from 4 to 6 weeks (*p* = 0.44). The baseline infected individuals had significantly higher ACE2 blocking antibodies than baseline uninfected individuals at 4 weeks (*p* < 0.0001) and at 6 weeks (*p* < 0.0001). The baseline infected individuals also developed significantly higher ACE2 blocking antibodies than those who were naturally infected (but unvaccinated) at 4 weeks (*p* < 0.0001) and at 6 weeks (*p* < 0.0001).

### Differences in the levels of antibodies to the RBD of the WT and SARS‐CoV‐2 variants

**FIGURE 2:**
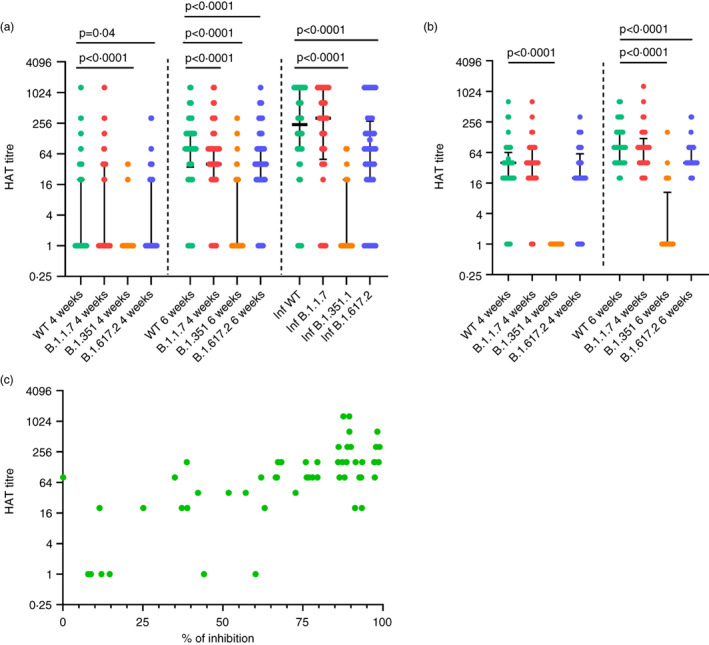
Antibodies to the RBD of SARS‐CoV‐2 Wuhan (WT) virus and the variants of concern by the haemagglutination test (HAT). (a) Antibodies to the RBD were measured by HAT in previously uninfected individuals at 4 weeks (*n* = 62) and at 6 weeks (*n* = 58), and in individuals who were naturally infected (*n* = 36) for the WT, alpha variant, beta variant and delta. (b) Antibodies to the RBD were also measured by HAT in previously infected individuals (*n* = 33), at 4 and 6 weeks to WT, alpha, beta and delta. The ACE2 receptor blocking antibody titres, were correlated with the HAT titre to the WT (Spearman's *r* = 0.67, *p* < 0.0001).The differences in HAT titres to WT and variants in baseline uninfected and infected individuals were assessed by the Wilcoxon matched pairs signed ranked test. All tests were two sided. the error bars indicate the median and the interquartile ranges. Green: WT; red: Alpha; orange: Beta; blue: Delta

Antibodies to the RBD were measured by HAT to the WT, B.1.1.7, B.1.351 and B.1.617.2 at 4 weeks (*n* = 62) and at 6 weeks (*n* = 58) in individuals who were baseline seronegative. The same cohort of individuals was used in for measuring ACE2 receptor blocking antibodies, HAT, T cell and B cell responses, although the number of individuals for some assays is less than that for the sVNT assays due to limited availability of sample volume for all assays. There was no difference between the HAT titres to the WT and alpha variant (*p* = 0.1) at 4 weeks, whereas levels were significantly lower to delta (*p* = 0.04) and beta variants (*p* < 0.0001) (Figure 2a). At 6 weeks (2 weeks after the second dose), the antibody levels were significantly lower (*p* < 0.0001) for alpha, beta and delta variants compared to responses to the WT. A HAT titre of 1:20 was considered as a positive result to the RBD by the HAT assay, as previously described [18]. At 4 weeks, 20 of 62 (32.2%) had a positive response to the WT, 22 of 62 (35.4%) to alpha, 3 of 62 (4.8%) to beta and 19 of 62 (30.6%) to delta variant. At 6 weeks (2 weeks after the second dose), 51 of 58 (87.9%) had a positive response to the WT, 50 of 58 (86.2%) to alpha, 16 of 58 (27.6%) for beta and 50 of 58 (86.2%) to delta variant. At 6 weeks, there was a 1.3‐fold reduction in the geometric means of the antibody titres to alpha (mean 132.4, SD ± 254.8) compared to the WT (mean 169.3, SD ± 258.4) 10.01‐fold reduction to beta (mean 16.9, SD ± 48.5) and a 1.38‐fold reduction to delta variant compared to the WT. There were no significant differences in the HAT titres to the WT, or variants between the different age groups at 4 weeks since receiving the first dose and at 6 weeks (Figure S2).

To compare the responses to the RBD of vaccinees with responses of convalescent sera, we analysed responses in 36 individuals who had varying severity of natural infection (onset of illness was 6 weeks before recruitment to the study). We found that those with natural infection had significantly less (*p* < 0.0001) HAT titres to beta and to delta than the WT but not alpha variant (Figure 2a). Those who were naturally infected had significantly higher HAT titres to the WT (*p* = 0.005) and alpha (*p* = 0.0002), than those who received the vaccine at 6 months. However, there was no significant difference for the HAT titres between those who were naturally infected, compared to the vaccinees for beta variant (*p* = 0.89) and delta (*p* = 0.13). Those who were naturally infected had 3.1‐fold higher geometric means of antibodies to RBD (HAT titres) to the WT, 3.9‐fold higher titres to alpha, no difference to beta and 2.3‐fold higher titres to delta variant.

Antibody responses were also assessed in baseline infected vaccinees at 4 weeks (*n* = 41) and at 6 weeks (*n* = 33, 2 weeks after the second dose). There was no significant difference in HAT titres to alpha (*p* = 0.30), and delta (*p* = 0.44), compared to the WT, but the levels were significantly lower to beta variant (*p* < 0.0001) at 4 weeks (Figure 2b). At 6 weeks, again there was no difference between the titres of WT and alpha (*p* = 0.15) but was significantly lower for beta (*p* < 0.0001) and delta (*p* < 0.0001). The HAT titres significantly increased to WT (*p* = 0.0004), alpha (*p* = 0.02), beta (*p* = 0.008) and delta variant (*p* = 0.003), following the second dose of the vaccine. After one dose, at 4 weeks, 38 of 41 (92.7%) had a positive response to WT, 38 of 41 (92.7%) to alpha, 0 of 41 (0%) to beta and 33 of 41 (80.5%) to delta. By 6 weeks, all 33 individuals had a positive response to the WT, alpha and delta, but only 8 of 33 (24.2%) had a positive response to beta variant (Figure 2b).

The ACE2 receptor blocking antibody titre, significantly correlated with the HAT titre to the WT (Spearman's *r* = 0.67, *p* < 0.0001) (Figure 2c), alpha (Spearman's *r* = 0.67, *p* < 0.0001), beta (Spearman's *r* = 0.38, *p* = 0.004) and delta variant (Spearman's *r* = 0.76, *p* < 0.0001) (data not shown).

### T cell responses to the Sinopharm/BBIBP‐CorV

**FIGURE 3:**
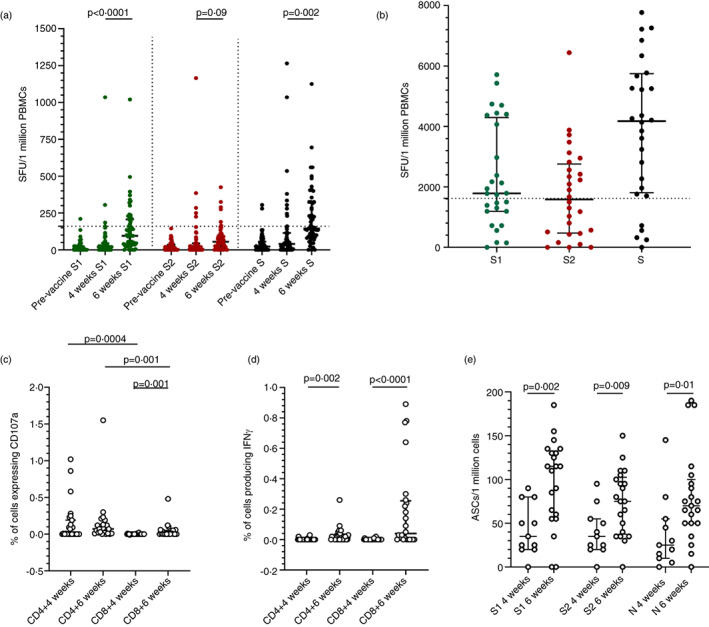
SARS‐CoV‐2 specific T cell and B cell responses in individuals who received the Sinopharm/BBIBP‐CorV vaccine. (a) Ex vivo IFNγ ELISpot assays were carried out at 4 weeks (*n* = 66) and 6 weeks (*n* = 58, 2 weeks after the second dose) for overlapping peptides of spike protein, which were in two pools S1 and S2. (b) Cultured IFNγ ELISpot assays were carried out at 6 weeks (*n* = 28), using the same pools. ICS was used to determine CD107a expression (c) and IFNγ production (d) at 4 weeks (*n* = 27) and at 6 weeks (*n* = 23) in CD4^+^ and CD8^+^ by flowcytometry. The number of antibody secreting cells (ASCs) were determined at 4 weeks (*n* = 11) and at 6 weeks (*n* = 21) by ELISpot assays after polyclonal in vitro activation of memory B cells. Wilcoxon matched pairs signed ranked test was used to find out the differences in ex vivo and cultured ELISpot responses for S1 and S2, and differences in CD107a expression and IFNγ production by CD4^+^ and CD8^+^ T cells at 4 and 6 weeks. All tests were two sided. the error bars indicate the median and the interquartile ranges. Green: S1 overlapping pool of peptides: Red; S2 pool overlapping pool of peptides; black: Overall magnitude of overlapping pools representing the spike protein

We investigated ex vivo IFNγ ELISpot responses in 66 individuals at 4 weeks and at 6‐week (*n* = 58). As a positive response was defined as mean ± 2 SD of the background responses, a cut‐off, of 160 SFU was considered as the threshold response. Accordingly at 4 weeks, 5 of 66 (7.6%) had responses to the S1 pool and 5 of 66 had responses to S2 (Figure 3a). At 6 weeks (2 weeks after the second dose), 18 of 66 (27.7%) had a positive response to S1 peptides, while 5 of 58 (8.6%) had responses to S2. The S1 specific responses at 4 weeks (median = 20, IQR = 0–45 SFU/1 million PBMCs) significantly increased (*p* < 0.0001) by 6 weeks (median = 95, IQR = 35–207.5 SFU/1 million PBMCs). However, there was no difference (*p* = 0.09) in responses to S2 at 4 weeks (median = 5, IQR = 0–43.7 SFU/1 million PBMCs) compared to those at 6 weeks (median = 55, IQR = 15–92.5 SFU/1 million PBMCs). There was no correlation of ex vivo IFNγ ELISpot responses to S1 or S2 or the total S, with age, at 4 weeks or at 6 weeks or with ACE2 receptor blocking antibodies or HAT RBD titres.

Cultured IFNγ ELISpot assays were carried out at 6 weeks (2 weeks since obtaining the second dose) in 28 individuals (only 28 were included due to limitation in cell numbers). As a positive response was defined as mean ± 2 SD of the background responses, a cut‐off, of 1620 SFU was considered as the response threshold. Accordingly, 13 of 28 had responses to S1, 11 of 28 had responses to S2 (Figure 3b). Although responses to S1 pool of peptides was higher than for S2, this was not significant (*p* = 0.09). There was no correlation between ex vivo and cultured IFNγ ELISpot responses at 6 weeks (Spearman's *r* = 0.15, *p* = 0.44). The ex vivo ELISpot responses to the S protein did not correlate with the ACE2 blocking antibodies (Spearman's *r* = 0.16, *p* = 0.23), with the HAT titres to the WT (Spearman's *r* = 0.19, *p* = 0.15), alpha (Spearman's *r* = 0.21, *p* = 0.16), beta (Spearman's *r* = −0.01, *p* = 0.98) or delta (Spearman's *r* = 0.19, *p* = 0.14).

ICS to assess CD107a expression and IFNγ production by CD4^+^ and CD8^+^ T cells was carried out at 4 weeks (*n* = 27) and at 6 weeks (*n* = 23) for the overlapping peptides of the spike protein. The CD4^+^ T cells demonstrated an increased membrane expression of CD107a than CD8^+^ T cells at 4 weeks (*p* = 0.0004) and 6 weeks (*p* = 0.001) (Figure 3c). Although, there was no significant difference in the membrane expression of CD107a from 4 weeks to 6 weeks for CD4^+^ T cells (*p* = 0.58) the expression significantly increased in CD8^+^ T cells (*p* = 0.002). At 4 weeks since the first dose, there was no IFNγ production detected by CD4^+^ T cells, and only CD8^+^ T cells of two individuals produced IFNγ (Figure 3d). IFNγ production by CD4^+^ T cells (*p* = 0.002) and CD8^+^ T cells (*p* < 0.0001), significantly increased from 4 to 6 weeks. In contrast to CD107a expression, CD8^+^ T cells (median = 0.04, IQR = 0%–0.25% of CD8^+^ T cells) produced significantly more (*p* = 0.02) IFNγ at 6 weeks than CD4^+^ T cells (median = 0.01, IQR = 0%–0.03% of CD4^+^ T cells).

### Memory B cell responses to Sinopharm/BB1BP‐CorV

To assess the levels of memory B cell, these were polyclonally stimulated in vitro and the frequency of antigen secreting plasmablasts were assessed by ELISpot assays at 4 weeks (*n* = 11) and at 6 weeks (*n* = 21). A positive response was defined as mean ± 2 SD of the background responses. Accordingly, a cut‐off of 62.9 antibody secreting cells (ASCs)/1 million cells was considered as the positive threshold for S1 protein, 35.7 for S2 and 47.7 for N at 4 weeks and 29.5 for S1, 38.8 for S2 and 35.4 ASCs/1 million cells for N at 6 weeks. At 4 weeks, 3 of 11 individuals had responses to S1, 5 of 11 for S2 and 2 of 11 for N. At 6 weeks, 19 of 21 had responses to S1, 16 of 21 for S2 and 17 of 21 for N protein. The ASC responses significantly increased from 4 to 6 weeks for S1 (*p* = 0.002), S2 (*p* = 0.009) and N (*p* = 0.01) (Figure 3e). The frequency of ASCs for S1 significantly correlated with ex vivo ELISpot responses to the second pool of overlapping peptides to the S protein (Spearman's *r* = 0.54, *p* = 0.01) but not with any ACE2 blocking antibodies or HAT titres against the WT of VOCs. The frequency of ASCs for S2 significantly correlated with HAT titres to the WT (Spearman's *r* = 0.46, *p* = 0.03) and alpha (Spearman's *r* = 0.57, *p* = 0.007), but not with ex vivo ELISpot responses or with ACE2 blocking antibodies.

## DISCUSSION

We investigated the SARS‐CoV‐2 specific total antibodies, ACE2 receptor blocking antibodies, antibody responses to the RBD of SARS‐CoV‐2 VOCs, ex vivo and memory T cell responses and functionality and memory B cell responses, in Sri Lankans following the Sinopharm/BBIBP‐CorV. As individuals are considered fully vaccinated, 2 weeks after obtaining the second dose of a COVID‐19 vaccines (for those which administer two doses) [26], the immune responses were assessed at 4 weeks after the first dose and 2 weeks after the second dose. Two weeks after obtaining the second dose of the vaccine, 95% of individuals seroconverted, although seroconversion rates were significantly lower in those who were >60 years (93.3%), compared to those in the 20–39 age group (98.9%). The seroconversion rates in this cohort were higher than reported in the phase 1 and 2 trials, probably as our cohort was larger [6].

Due to limitations, we used a surrogate neutralizing test, which has shown to correlate well with neutralizing antibodies [13]. We found that by 6 weeks (2 weeks following the second dose), 81.25% of individuals had ACE2 receptor blocking antibodies, which was lower than reported in the phase 1 and 2 trials, which used live virus assays [6]. However, the ACE2 receptor blocking antibody titres 2 weeks after the second dose of the vaccine, was similar to the levels seen in convalescent sera. Therefore, the vaccine appears to induce a similar level of ACE2 receptor blocking antibodies as following natural infection.

Both convalescent sera and sera from individuals who received the Sinopharm/BBIBP‐CorV vaccine had reduced neutralizing capacity of delta variant and to a greater extent to beta [27]. The vaccinees had a significant reduction of RBD binding antibodies the WT and the alpha variant compared to naturally infected, but not for delta variant and beta variant. This suggests that the vaccinees had a similar level of protection against infection with delta and beta, as those who were naturally infected. The vaccinees only had a 1.38‐fold reduction in the RBD binding antibodies to delta variant compared to the WT, whereas a 10‐fold reduction was seen against beta variant, suggesting that the Sinopharm/BBIBP‐CorV is likely to have higher efficacy to delta variant than for beta variant. Since the reduction in the geometric means of the RBD antibodies to delta was only 1.38 fold less than for the WT, the reduction in vaccine efficacy is likely to be less in countries where delta is the predominant variant. Therefore, it is important to assess the vaccine efficacy against delta by carefully conducted post‐vaccine surveillance studies.

We investigated the T cell and B cell responses to the Sinopharm/BBIBP‐CorV vaccinated individuals in this study. We found that 27.7% of individuals had ex vivo IFNγ ELISpot responses in high frequency to the spike protein overlapping peptides. However, the magnitude of responses and the number of individuals who had responses were less than for a single dose of the AZD1222 vaccine [17], which shown to induce high frequency and magnitude of polyfunctional T cell responses even after a single dose [3, 28]. Sinopharm/BBIBP‐CorV was show to induced potent IFNγ responses as detected by ICS from CD8^+^ T cells, 2 weeks following the second dose, although degranulation (membrane expression of CD107a) responses predominantly seen from the CD4^+^ T cell subset. The CD107a expression by CD8^+^ T cells after the second dose of Sinopharm/BBIBP‐CorV were similar to those observed after a single dose of the AZD1222 [3]. As Sinopharm/BB1B‐CorV is a whole cell inactivated vaccine, individuals are also likely to develop T cell responses to other proteins such as the nucleocapsid protein of the virus, which we did no assess in this study. While some studies have shown that a high magnitude of T cell responses to spike, nucleocapsid and membrane proteins in patients with acute COVID‐19 was not associated with recovery [29], T cell responses against certain epitopes of the N protein is shown to result in less severe disease [30]. As we only studies T cell responses specific for the spike protein, it is likely that we did not capture the breadth of the T cell responses in those who received this vaccine.

In summary, 95% of individuals appear to seroconvert following the Sinopharm/BBIBP‐CorV vaccine, and the vaccine appears to induce similar levels of ACE2 receptor blocking antibodies and RBD binding antibodies to delta variant as seen following natural infection; 27.7% of individuals had ex vivo IFNγ ELISpot responses to the vaccine, while CD107a expression was predominantly seen from the CD4^+^ subset of T cells. However, seroconversion rates and immunogenicity appear to be lower in older individuals.

## CONFLICT OF INTEREST

The authors declare no conflict of interest.

## ETHICS STATEMENT

Ethical approval was received by the Ethics Review Committee of Faculty of Medical Sciences, University of Sri Jayewardenepura. Informed written consent was obtained from patients. All individuals who participated in the study gave informed written consent.

## Supporting information


**Figure S1** SARS‐CoV‐2 CE2 receptor blocking antibodies in those who received the Sinopharm/BBIBP‐CorV vaccine. ACE2 receptor blocking antibodies were measured by the surrogate neutralizing antibody assay in individuals who were 20–39 (*n* = 21), 40–59 (*n* = 31) and >60 (*n* = 12) at 4 weeks and at 6 weeks. The differences in the total antibody titres between different age groups was determined by the Kruskal–Wallis test. All tests were two sided. The error bars indicate the median and the interquartile ranges.
**Figure S2** Antibodies to the RBD of SARS‐CoV‐2 Wuhan (WT) virus and the variants of concern by the haemagglutination test (HAT) in individuals of different age groups. Antibodies to the RBD were measured by HAT for WT, B.1.1.7, B.351.1 and B.1.617.2 in previously uninfected individuals who were 20–39 (*n* = 20), 40–59 (*n* = 27) and >60 (*n* = 11) at 6 weeks (2 weeks since receiving the second dose of the vaccine). The differences in the HAT titres between different age groups was determined by the Kruskal–Wallis test. All tests were two sided. The error bars indicate the median and the interquartile ranges.

**Appendix S1** Supporting Information

## Data Availability

All data is available in the manuscript and the figures.
