# Supplementary material for: Immune responses to Sinopharm/BBIBP‐CorV in individuals in Sri Lanka
Source: Immunology. 2022 Jul 12;167(2):275–85. doi: 10.1111/imm.13536 (PMC11495257; doi:10.1111/imm.13536)
Supplement: Supplementary file 1 — Figure S1 SARS‐CoV‐2 CE2 receptor blocking antibodies in those who received the Sinopharm/BBIBP‐CorV vaccine. ACE2 receptor blocking antibodies were measured by the surrogate neutralizing antibody assay in individuals who were 20–39 (n = 21), 40–59 (n = 31) and >60 (n = 12) at 4 weeks and at 6 weeks. The differences in the total antibody titres between different age groups was determined by the Kruskal–Wallis test. All tests were two sided. The error bars indicate the median and the interquartile ranges. Figure S2 Antibodies to the RBD of SARS‐CoV‐2 Wuhan (WT) virus and the variants of concern by the haemagglutination test (HAT) in individuals of different age groups. Antibodies to the RBD were measured by HAT for WT, B.1.1.7, B.351.1 and B.1.617.2 in previously uninfected individuals who were 20–39 (n = 20), 40–59 (n = 27) and >60 (n = 11) at 6 weeks (2 weeks since receiving the second dose of the vaccine). The differences in the HAT titres between different age groups was determined by the Kruskal–Wallis test. All tests were two sided. The error bars indicate the median and the interquartile ranges. [file IMM-167-275-s002.docx]

**Supplementary figures**


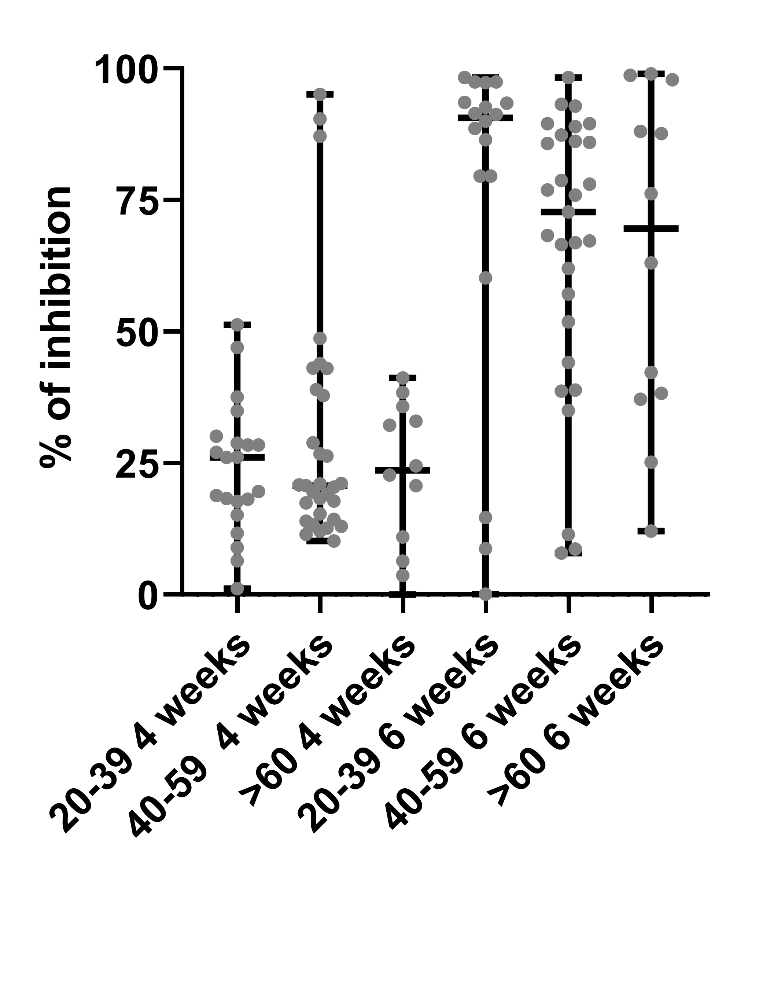


**Supplementary figure 1:** **SARS-CoV-2 CE2 receptor blocking antibodies in those who received the Sinopharm.BBIBP-CorV vaccine.** ACE2 receptor blocking antibodies were measured by the surrogate neutralizing antibody assay in individuals who were 20 to 39 (n=21), 40 to 59 (n=31) and >60 (n=12) at 4 weeks and at 6 weeks. The differences in the total antibody titres between different age groups was determined by the Kruskal-Wallis test. All tests were two sided. The error bars indicate the median and the interquartile ranges.


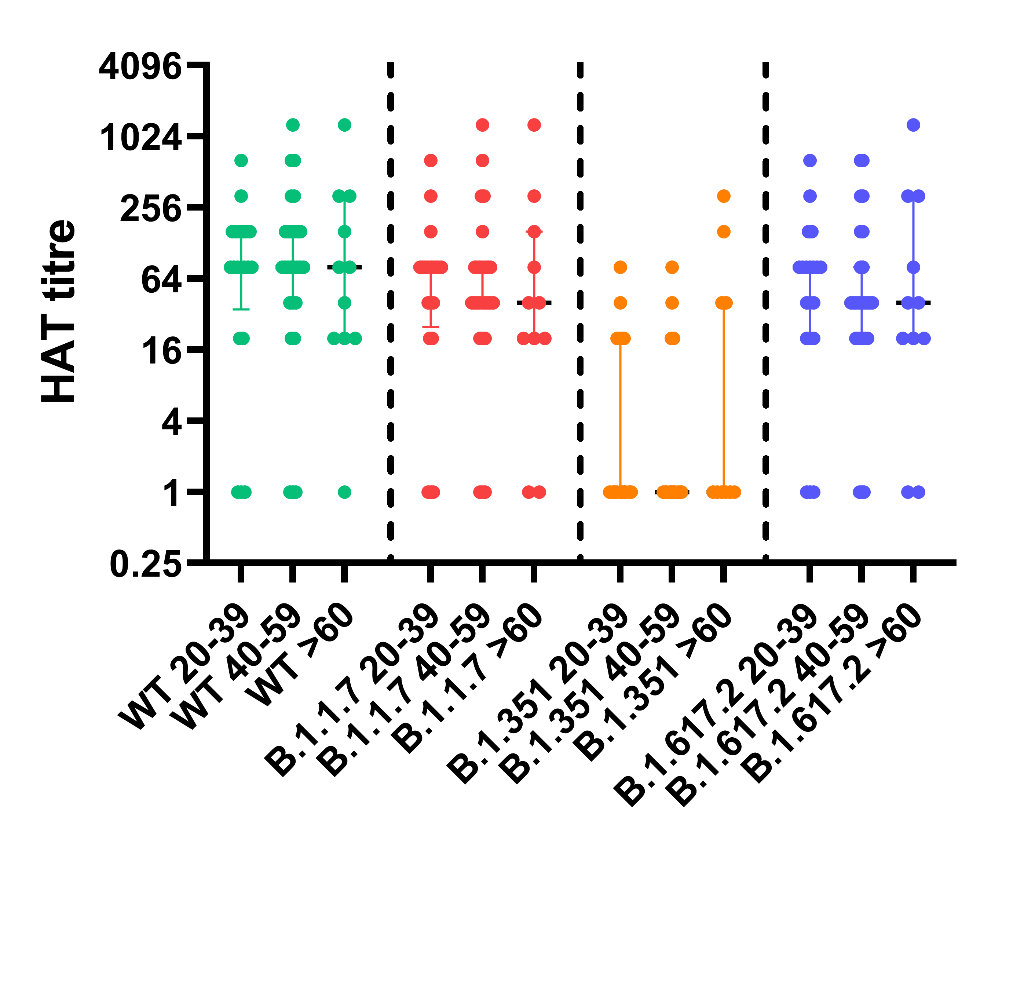


**Supplementary figure 2: Antibodies to the RBD of SARS-CoV-2 Wuhan (WT) virus and the variants of concern by the haemagglutination test (HAT) in individuals of different age groups.** Antibodies to the RBD were measured by HAT for WT, B.1.1.7, B.351.1 and B.1.617.2 in previously uninfected individuals who were 20 to 39 (n=20), 40 to 59 (n=27) and >60 (n=11) at 6 weeks (2 weeks since receiving the second dose of the vaccine). The differences in the HAT titres between different age groups was determined by the Kruskal-Wallis test. All tests were two sided. The error bars indicate the median and the interquartile ranges.
