# Supplementary material for: Immune responses to Sinopharm/BBIBP‐CorV in individuals in Sri Lanka
Source: Immunology. 2022 Jul 12;167(2):275–85. doi: 10.1111/imm.13536 (PMC11495257; doi:10.1111/imm.13536)
Supplement: Supplementary file 2 — Appendix S1 Supporting Information [file IMM-167-275-s001.docx]

**Supplementary methods**

**PBMC separation**

Whole blood 10ml was collected from donors into either heparin- or EDTA-containing 50ml tube. Blood was diluted with an equal volume of RPMI1640 and layered on top of the lymphoprep (Alere technologies cat 1114547) 15ml containing tube. The tubes were centrifuged at 2000rpm without breaks 20 min at 20°C, and the buffy layer (cloudy layer) containing the PBMCs was removed. PBMCs was re-suspended in 20ml of RPMI1640 and centrifuged at 1600rpm 10 min with breaks at 20°C. Previous step was repeated. Cell pellet was re-suspended in 1ml of R10 media and cell count was taken.

**Media preparation**

T cell culture media

1ml of penicillin/ streptomycin antibiotic (Thermofisher (USA) Cat: 15140122) and 1ml of glutamine (Thermofisher (USA) Cat: 25030081) were added to 98ml of RPMI1640 medium (Sigma Aldrich (Germany) Cat: R8758) and filtered using syringe filter (0.2µm pore size).

HR10 media

10ml of heat inactivated human serum (Sigma Aldrich (Germany) Cat: H3667) was added to 90 ml of T cell culture media and filtered using syringe filter (Millipore (Germany) Cat: SLGP033RS) (0.2µm pore size).

Feeding media

100µl of IL-2 (Peprotech (USA) Cat: 200-02) (1 X 10^5^ units/ml) was added to 50ml of HR10 media and filtered using syringe filter (Millipore (Germany) Cat: SLGP033RS) (0.2µm pore size).

R10 media

10ml of heat inactivated fetal bovine serum (FBS) (Sigma Aldrich (Germany) Cat: F4135) was added to 90ml of T cell culture media and filtered using syringe filter (Millipore (Germany) Cat: SLGP033RS) (0.2µm pore size).

***Ex vivo* ELISpot assay procedure**

Thirty five percent ethanol 100µl/well was added to ELISpot plate (96 well plate) and keep 1min. Ethanol was discarded and wells were washed 5 times using 200µl of sterile distil water. Plate was dried using a paper towel. Capture monoclonal antibody (1-D1K) 75µl was diluted in 5ml of sterile PBS and mix well, 50µl of antibody solution was added to each well. Plate was covered using parafilm and store at 4°C overnight.

ELISpot plate was washed 4 times using 150µl/well of RPMI1640. R10 medium 100µl/well was added and incubated in 5% CO2 incubator at 37°C 1 hour. Freshly isolated PBMC 100,000/well were added, which was prepared as cell suspension in R10 150µl, S1 and S2 peptide pools of wild type SARS-CoV-2 virus were added at a final concentration of 10 μM to two adjacent wells and mixed. Phytohaemagglutinin (PHA) (Sigma Aldrich, USA) (20µl of PHA) was used as a positive control and peptides were not added to negative control (cells with media) (figure 1). Experiments were carried out in duplicates. Plate was incubated in 5% CO2 incubator at 37°C overnight.

After removing the cells, ELISpot plate was washed 6 times using 220µl/well PBST. Biotinylated detection monoclonal antibody (7-B6-1) 10µl was diluted in 10ml of sterile PBS. ELISpot plate was dried using paper towel and add Biotinylated detection monoclonal antibody (7-B6-1) solution 100µl/well. Plate was incubated 2 hours at room temperature. Content of the plate was discarded and wash 6 times using 220µl/well PBST. Streptavidin-ALP 10µl was diluted in 10ml of sterile PBS. ELISpot plate was dried and add Streptavidin-ALP solution 100µl/well. Plate was incubated 1 hours at room temperature. NBT substrate 10ml was filtered using 0.22µm syringe filter. Content of the plate was discarded and washed 6 times using 220µl/well PBST. Dried the plate and add NBT substrate 100µl/well. Plate was incubated 10min at room temperature in a dark place. Content was discarded and washed the plate under the running tap water. Bottom layer of the plate was removed and kept in a dark place at room temperature for one week. ELISpot plate was read by using ELISpot reader (AID Germany). Background (PBMCs plus media alone) was subtracted and data expressed as number of spot-forming units (SFU) per 10^6^ PBMCs. A positive response was defined as mean±2 SD of the background responses.


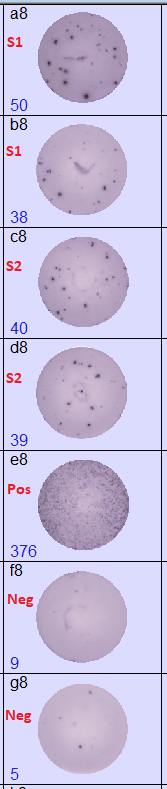


Supplementary methods Figure 1: An example of an ex vivo ELISpot response at 6 weeks (2 weeks following the second dose of the vaccine).

**Cultured ELISpot assay**

The PBMC suspension was prepared by using HR10 medium, final cell density was 2.0 X 10^6^ cells/ml, this cell suspension was added to two wells of 24 well plate (2ml/well). Cells were stimulated by adding 200µl of 40µM spike protein. Plate was incubated 3 days in 5% CO2 incubator at 37°C. Volume of 1ml was removed from the top of the well, and 1 ml of fresh feeding media was added to the well and mix. Plate was incubated 4 days in 5% CO2 incubator at 37°C. Cells were fed and incubated 3 days in 5% CO2 incubator at 37°C. Tenth day cell suspension was taken into sterile 50ml tube and top up to 20ml by using RPMI1640, centrifuged the tube at 363 X g 10 min with breaks at 20°C. Cell pellet was re-suspended in 20ml of RPMI1640 and centrifuge the tube at 363 X g 10 min with breaks at 20°C. Cell pellet was re-suspended in 4ml of HR10 and add to two new wells of 24 well plate (2ml/well). Plate was incubated in 5% CO2 incubator at 37°C overnight.

Human IFN-gamma ELISpot BASIC (ALP) (3420-2A) ELISpot kit (Mabtech, Sweden) was used for this experiment. Polyvinylidene difluoride (PVDF) membrane of the 96 well ELISpot plate (MAIPS4510 Millipore MultiScreen-IP Filter Plate, USA) was treated with 35% ethanol and kept 1min. Ethanol was discarded and wells were washed 5 times using 200µl of sterile distil water. Plate was dried using paper towel. Plate was coated with anti-human IFNγ antibody (Capture monoclonal antibody (1-D1K), Mabtech, Sweden) and incubated overnight at 4°C. Briefly, IFNγ was used at 15µl per 1ml of sterile phosphate-buffered saline (PBS) and 50µl of this was added to each well. Paraffin was used to wrap the plate to prevent evaporation of antibody solution.

Cells from 24 plate wells were washed on day 10 with RPMI 1640. The cells were rested for 1 to 2 days before testing. After overnight incubation of ELISpot plate, the excess antibody was removed by washing 4 times with 150 μl/well RPMI. The plate was then blocked by adding R10 (100 μl/well) and incubate at 37°C 5% CO2 for 1 hour. PBMC of 24 well plate was taken into 50ml sterile tube and topped to up 20ml by using RPMI1640, centrifuged at 363 X g 10 min with breaks at 20°C. Cell pellet was resuspended in 1ml of R10 and cell count was taken. Cell suspension was prepared in 1.5ml of R10 medium by adding 400,000 PBMCs. ELISpot plate was washed 5 times using 200µl/well RPMI1640 and single wash using sterile distilled water 200µl/well. Plate was dried by using paper towel, cell suspension was added 150µl/well (40000 cells/well). S1 and S2 peptide pools (two pools of overlapping peptides named S1 (peptide 1 to 130) and S2 (peptide 131 to 253) covering the whole spike protein (253 overlapping peptides. All peptide sequences were derived from the wild-type consensus) 50µl (40µmol) were added to two adjacent wells as duplicates and mix (final concentration of the peptide was 10μM) (Mongkolsapaya *et al.*, 2006;Malavige *et al.*, 2008). Phytohaemagglutinin (PHA) (Sigma Aldrich, USA) (20µl of PHA at 40µM) was used as a positive control (Pos), peptides were not added to negative control (Neg) (Figure 2). Plate was incubated in 5% CO2 incubator at 37°C overnight.

**
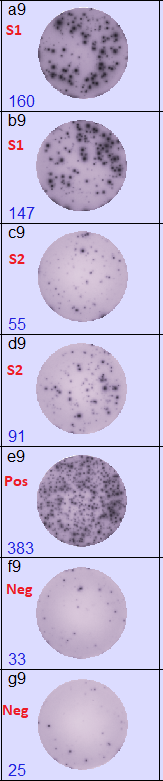
**

Supplementary methods Figure 2: An example of a cultured ELISpot response at 6 weeks (2 weeks following the second dose of the vaccine).

**ELISpot plate development**

Removed the cells and ELISpot plate was washed 6 times using 220µl/well PBST. Biotinylated detection monoclonal antibody (7-B6-1, Mabtech, Sweden) 10µl was diluted in 10ml of sterile PBS. ELISpot plate was dried using paper towel and added Biotinylated detection monoclonal antibody (7-B6-1) solution 100µl/well. Plate was incubated 2 hours at room temperature. Content of the plate was discarded and washed 6 times using 220µl/well PBST. Streptavidin-ALP 10µl was diluted in 10ml of sterile PBS. ELISpot plate was dried and added Streptavidin-ALP solution 100µl/well. Plate was incubated 1 hours at room temperature. NBT substrate 10ml was filtered using 0.22µm syringe filter. Content of the plate was discarded and washed 6 times using 220µl/well PBST. Dried the plate and add nitrobluetetrazolium–5-bromo-4-chloro-3-indolylphosphate (BCIP/NBT) substrate (Mabtec, Sweden) substrate 100µl/well. Plate was incubated 10min at room temperature in a dark place. Content was discarded and wash the plate under the running tap water. Bottom layer of the plate was removed and kept in a dark place at room temperature for one week. ELISpot plate was read by using ELISpot reader (AID Germany). Background (PBMCs plus media alone) was subtracted and data expressed as number of spot-forming units (SFU) per 10^6^ PBMCs. A positive response was defined as mean±2 SD of the background responses.


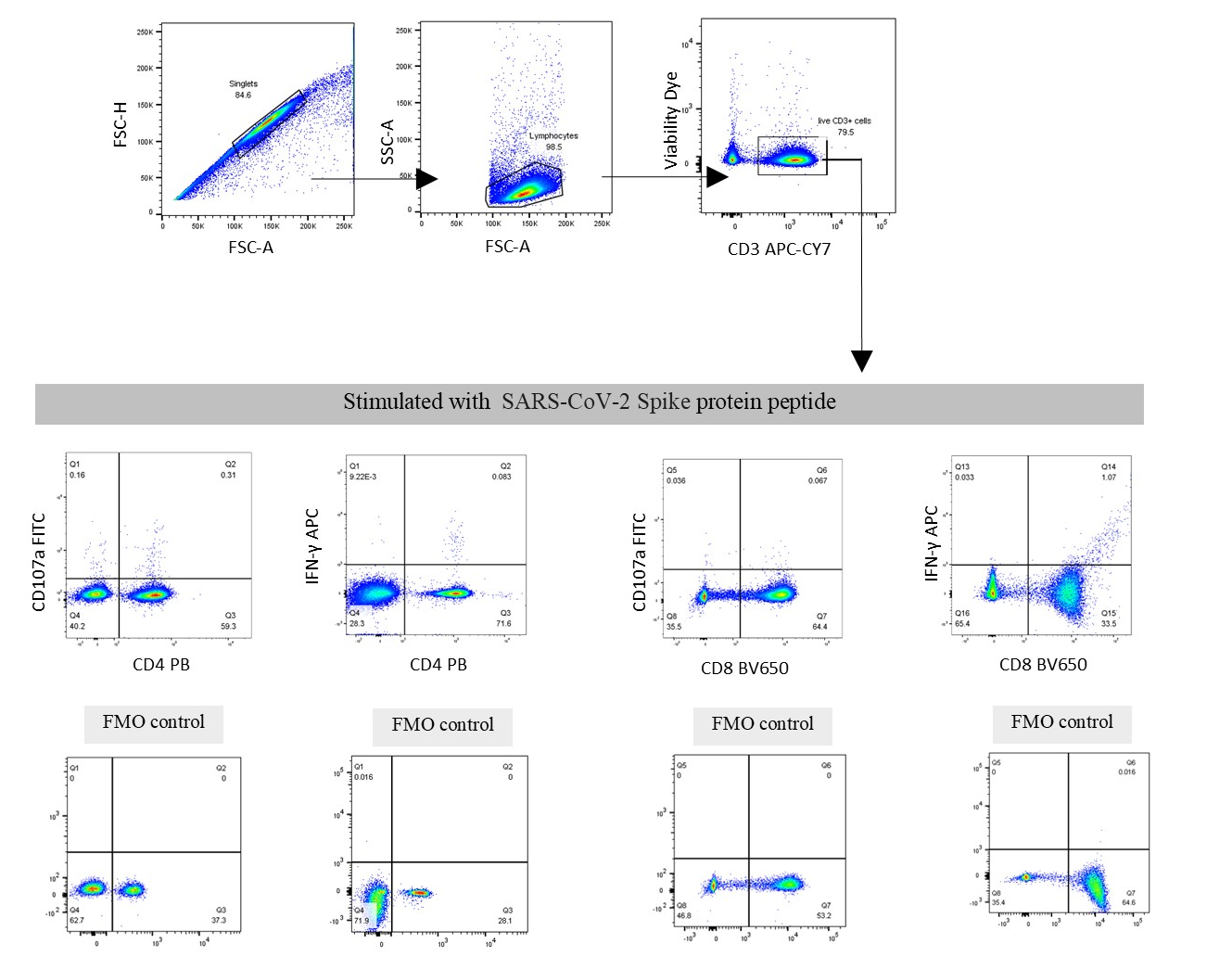


**Supplementary methods figure 3:** Gating strategy used to identify CD107a expressing CD4+ and CD8+ T cells and IFNγ producing CD4+ and CD8+ T cells. The cells were initially gates on FSC-H and FSC-A to gate the singlets. The lymphocytes from these cells were then identified by gating them on the FSC and SSC. From these cells, the live cells were then gated and then CD3+ T cells were gated. From these CD3+ T cells, CD107a expressing CD4+ and CD8+ T cells were identified, and IFNγ producing CD4+ T cells and CD8+ T cells were identified.

B cell ELISpot assays

Briefly, freshly isolated PBMCs were stimulated in a 24 well plate using IL-2 and R848 (a TLR 7/8 agonist) in RPMI supplemented with 10% fetal bovine serum, 1% penicillin streptomycin and 1% glutamine at 4 million cells/well and incubated at 37 °C with 5% CO2 for 3 days. They were then washed and rested overnight and 100,000 cells/well were added. 50,000 cells/well were added to the positive control wells. A Human IgG ELISpot kit (Mabtech 3850-2A) was used according to the manufacturer’s instructions to quantify IgG-secreting cells specific to SARS-COV2 S1, S2 and N recombinant proteins, which were coated at 2µg/ml in phosphate buffered saline (PBS). All experiments were carried out in duplicate and anti-human IgG monoclonal capture antibodies, was used as a positive control, and media alone as a negative control. The spots were enumerated using an automated ELISpot reader (AID Germany). A positive response was defined as mean±2 SD of the background responses. An example of a B cell ELISpot assay for S1, S2 and N recombinant proteins is shown in supplementary methods figure 4.


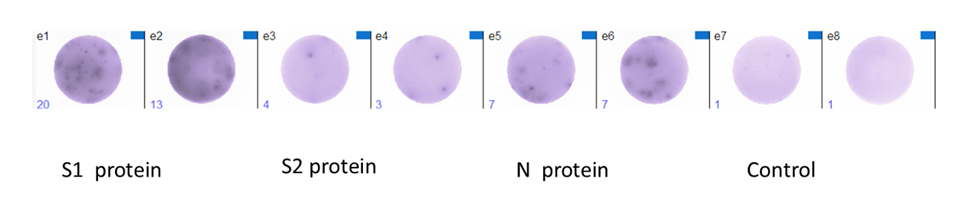


Supplementary methods figure 4: An example of a B cell ELISpot assay at 6 weeks (2 weeks since administering the second dose) for S1, S2 and N recombinant protein.
